# Acute Recurrent Pancreatitis and COVID-19 Infection: A Case Report with Literature Review

**DOI:** 10.7759/cureus.13490

**Published:** 2021-02-22

**Authors:** Harindra Sandhu, Dhiraj Mallik, Manoj Joshua Lokavarapu, Farhanul Huda, Somprakas Basu

**Affiliations:** 1 General Surgery, All India Institute of Medical Sciences, Rishikesh, IND; 2 General Surgery, Lala Lajpat Rai Memorial Medical College, Meerut, IND

**Keywords:** covid-19, acute recurrent pancreatitis, pancreatitis

## Abstract

Severe acute respiratory syndrome coronavirus 2 (SARS-CoV-2) infection typically presents with respiratory symptoms, although presentation with gastrointestinal symptoms is not uncommon. Coronavirus disease 2019 (COVID-19) presenting as acute pancreatitis is rare. There are several etiological factors for acute recurrent pancreatitis, but its association with COVID-19 disease is not yet known. We present an unusual case of recurrent attacks of acute pancreatitis in a young woman with SARS-CoV-2 infection, which was diagnosed early but had a rapid downhill course in the second attack with a fatal outcome.

## Introduction

Severe acute respiratory syndrome coronavirus 2 (SARS-CoV-2) infection typically presents with respiratory symptoms and acute pancreatitis as a presentation is rare with few case reports [1-5]. Hypoxia, acute respiratory distress syndrome, atelectasis, and pleural effusion are known pulmonary complications in acute pancreatitis and severe SARS-CoV-2 infection [6]. Therefore, diagnosis of coronavirus disease 2019 (COVID-19) infection as a cause of acute pancreatitis may be overlooked clinically.

Recurrent attacks of acute pancreatitis within few months due to COVID-19 are not yet known. In pulmonary complications due to acute pancreatitis, COVID-19 may not be thought of as an etiological factor. We present an unusual case of acute recurrent pancreatitis in a young woman with recent SARS-CoV-2 infection, who had a declining course in the second attack with a fatal outcome.

## Case presentation

A 25-year-old woman with known diabetes mellitus on irregular treatment presented to the emergency department with complaints of upper abdominal pain for the last seven days and high grade fever and acute shortness of breath for the last two days. Upper abdominal pain was of sudden onset, severe in intensity. Fever was associated with chills and was only relieved with antipyretics. Shortness of breath was sudden in onset which was not associated with cough, sore throat, or chest pain. There was a similar history of abdominal pain 1.5 months back for which she was treated in the intensive care unit with a diagnosis of acute pancreatitis. She was discharged after nine days without any residual sequel. She denied any history of smoking, alcoholism, history of gallstone disease, any specific drug history (apart from diabetes treatment), or family history of similar disease. 

**Figure 1:**
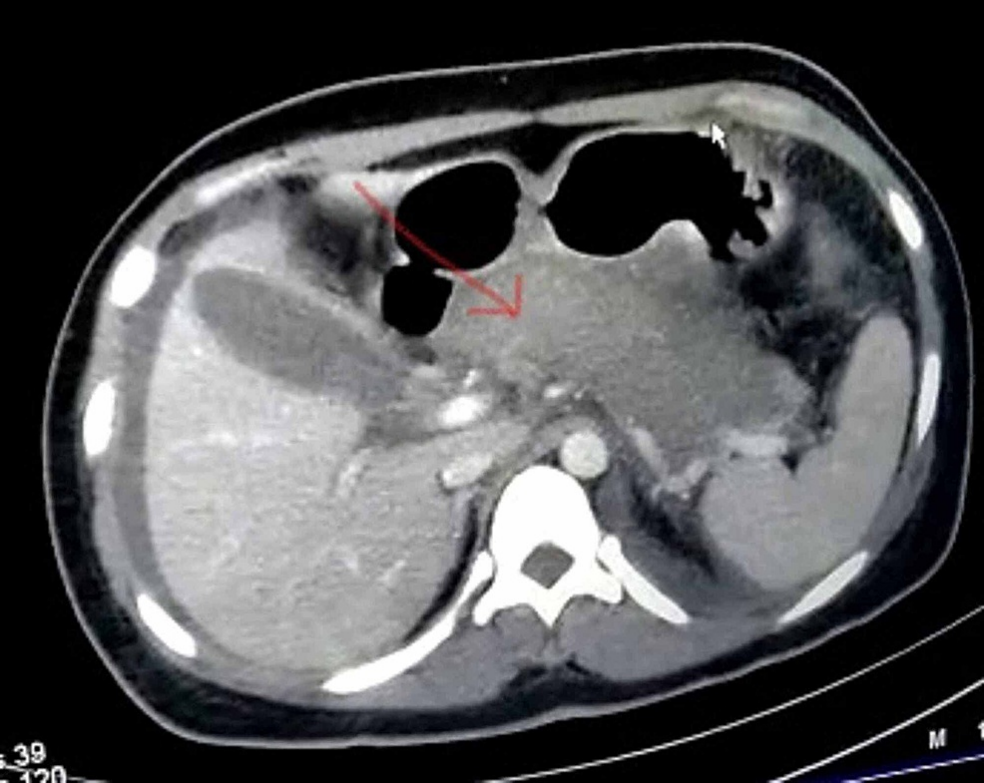
Contrast-enhanced computed tomographic scan showing diffuse enlargement of pancreas with hypo-enhancement (red arrow)

Her physical examination revealed a pulse rate of 98/min, blood pressure of 110/70 mmHg, and respiratory rate of 22/min. An abdominal examination revealed a tender area with muscle guard in the epigastric region. Her random blood sugar was 343 mg/dL, oxygen saturation (SpO2) of 85% on room air and 98% on face mask with an oxygen reservoir at 12 litres per minute, and decreased air entry on the left side. Blood investigations revealed hyperamylasemia (350 U/L), low hemoglobin (8.9 g/dl), high international normalized ratio (INR) (2.03), high serum lipase (35.6 U/L), and raised liver enzymes (serum glutamic pyruvic transaminase (SGPT): 79 U/L, serum glutamic oxaloacetic transaminase (SGOT): 163 U/L). There was no evidence of hypertriglyceridemia or hypercalcemia. Her arterial blood gas analysis revealed type I respiratory failure, and chest X-ray showed left-sided pleural effusion with underlying basal consolidation. A contrast-enhanced CT scan of whole abdomen showed features of acute pancreatitis (modified CT severity score of 6) (Figure 1).

There was no evidence of gallstones or bile duct stones, pancreatic developmental anomaly, or radiological evidence of chronic pancreatitis.

Initially, she was admitted to the COVID suspect ward and was later shifted to the COVID ICU after her nasal and oropharyngeal swab for SARS-CoV-2 reverse transcriptase-polymerase chain reaction (RT-PCR) tested positive. She was managed with bowel rest, high flow oxygen, intravenous crystalloid fluid resuscitation, IV analgesic, empirical antibiotics, human regular insulin, and vitamin K injection. After COVID-19 positive report, low molecular weight heparin and low dose dexamethasone were started. On second day, she developed hypotension, which was managed initially by IV fluids and later by ionotropic support. She became febrile, and her respiratory distress worsened with progressive hypoxia refractory to high flow oxygen for which she had to be intubated and kept on mechanical ventilation. Her condition deteriorated, and she had a cardiac arrest and could not be revived on the fourth day of admission.

## Discussion

COVID-19 disease also has its impact on the gastrointestinal tract in less than 10% of cases which include nausea/vomiting (7.8%), diarrhea (7.7%), abdominal pain (2.7%), and liver enzyme abnormalities (15%) [7]. Initially, there appeared to be no link between the SARS-CoV-2 virus and the pancreas. Since then, a few cases of COVID-19 disease presenting as acute pancreatitis have been reported. Several mechanisms of pancreatic injury have been described, such as direct cytopathic effect of the virus and dysregulated immune response induced by SARS-CoV-2 that targets the pancreas in addition to the lungs [1-5].

COVID-19 disease may present with elevation of serum amylase and lipase without radiological features of pancreatitis [5], which may attribute to increased replication of viruses in the pancreas. Conversely, the rise in enzymes can also attribute to inflammation of non-pancreatic origin. In this present case, SARS-CoV-2 was an antecedent cause of acute pancreatitis by direct cytopathic injury or whether an attack of acute pancreatitis with its systemic inflammatory state predisposed the patient to SARS-CoV-2 virus is a matter of debate. The patient survived the first attack, but the second one proved to be fatal. Her uncontrolled diabetes mellitus probably accentuated the inflammatory course. It is not known whether she was COVID-19 positive in the first attack as COVID test was not done at that time. So a possibility of first attack due to viral infection cannot be ruled out. Rapid downhill course of the disease due to overwhelming inflammation suggests that the virus was the cause of pancreatitis. It is known that recurrent infections with a similar virus over a short period may be unduly fatal [8].

Acute recurrent pancreatitis refers to repeated episodes of acute pancreatitis, diagnosed retrospectively. Several etiological factors (mechanical, inherited, autoimmune, metabolic, drugs, parasites, vascular disorders, and toxic substances) can lead to recurrent acute episodes. However, 30% of cases do not have a known etiology and are diagnosed as idiopathic [9]. The relationship between acute recurrent pancreatitis and viral etiology has not been reported, but the present case scenario may be a pointer to this possibility.

Acute pancreatitis can be caused by many viruses such as hepatitis B virus and Coxsackie B virus [10]. Clinical features and the natural course of pancreatitis caused by SARS-CoV-2 are not well established. The scoring system and criteria for diagnosis in COVID-19 induced pancreatitis are also not known. It appears from the present case and from those reported previously, that SARS-CoV-2 infection-induced acute pancreatitis leads to accelerated organ failure due to a more aggressive inflammatory response. In the present scenario, we highlight the fact that in all cases of acute pancreatitis, COVID-19 RT-PCR should be done. It would not be out of place to consider that patients who recover from acute pancreatitis with suspected or proven COVID-19 infection need to be followed up closely as a second attack might be life-threatening.

## Conclusions

SARS-CoV-2 virus can cause exaggeration of symptoms in pancreatitis patients by accelerating the inflammatory response (organ failure) and ending up with a high rate of mortality within a short span of time. While acute recurrent pancreatitis caused by SARS-CoV-2 virus is a possibility, SARS-CoV-2 induced acute pancreatitis should be closely followed up after discharge for any similar recurrent symptoms.
